# Premenopausal endogenous oestrogen levels and breast cancer risk: a meta-analysis

**DOI:** 10.1038/bjc.2011.358

**Published:** 2011-09-13

**Authors:** K Walker, D J Bratton, C Frost

**Affiliations:** 1 Faculty of Epidemiology and Population Health, London School of Hygiene and Tropical Medicine, Keppel Street, London WC1E 7HT, UK; 2 MRC Clinical Trials Unit, 125 Kingsway, London WC2B 6NH, UK

**Keywords:** breast cancer, oestrogen, premenopausal, hormones, meta-analysis

## Abstract

**Background:**

Many of the established risk factors for breast cancer implicate circulating hormone levels in the aetiology of the disease. Increased levels of postmenopausal endogenous oestradiol (E2) have been found to increase the risk of breast cancer, but no such association has been confirmed in premenopausal women. We carried out a meta-analysis to summarise the available evidence in women before the menopause.

**Methods:**

We identified seven prospective studies of premenopausal endogenous E2 and breast cancer risk, including 693 breast cancer cases. From each study we extracted odds ratios of breast cancer between quantiles of endogenous E2, or for unit or s.d. increases in (log transformed) E2, or (where odds ratios were unavailable) summary statistics for the distributions of E2 in breast cancer cases and unaffected controls. Estimates for a doubling of endogenous E2 were obtained from these extracted estimates, and random-effect meta-analysis was used to obtain a pooled estimate across the studies.

**Results:**

Overall, we found weak evidence of a positive association between circulating E2 levels and the risk of breast cancer, with a doubling of E2 associated with an odds ratio of 1.10 (95% CI: 0.96, 1.27).

**Conclusion:**

Our findings are consistent with the hypothesis of a positive association between premenopausal endogenous E2 and breast cancer risk.

Many breast cancer risk factors are believed to operate through circulating hormone levels; for example, early menarche, late menopause, fewer full-term pregnancies and delayed first full-term pregnancy. Specifically, early menarche and late menopause implicate oestrogens, progesterone, or both in the aetiology of breast cancer (Travis and Key, 2003). Incidence of breast cancer is seen to increase sharply with age in premenopausal women, but following the menopause, when oestrogen and progesterone levels both drop, the rate of increase in risk with age is dramatically reduced (Pike *et al*, 1993). The main mechanisms hypothesised to explain how circulating hormones could increase breast cancer risk are increased cell proliferation and mutagenesis (Pike *et al*, 1993; Travis and Key, 2003; Russo and Russo, 2004; Martin and Boyd, 2008). Oestradiol (E2) has been shown to increase breast cell mitosis *in vitro* (Key, 1999), and oestrogens and their metabolites have been demonstrated to induce DNA damage, genetic instability and cell mutations both in culture and *in vivo* (Travis and Key, 2003). Higher levels of endogenous oestrogens have been shown to be associated with an increased risk of breast cancer in postmenopausal women; a pooled analysis of nine prospective studies found that a doubling in the levels of E2 increased a woman's odds of developing breast cancer by 29% (95% CI: 15, 44%) (The Endogenous Hormones and Breast cancer Collaborative Group, 2002). However, no such association has been established in premenopausal women, possibly because of difficulties in measuring premenopausal E2, which has cyclic variation throughout the menstrual cycle (Key, 1999; Travis and Key, 2003).

The evidence on endogenous premenopausal oestrogens and breast cancer risk is mixed. Most studies have been fairly small, and the largest study to date, which included around 300 breast cancer cases, showed no evidence of an association. Breast cancer is rare in women at young ages, which makes it difficult to accumulate large numbers of cases prospectively. In addition, the within-subject variation in oestrogen levels is greater in premenopausal than postmenopausal women, giving rise to a greater degree of attenuation of effects through regression dilution bias, and this consequently limits the statistical power of individual small studies. This is a strong motivation for a meta-analysis: by pooling the estimates, the effective sample size is increased and effects can be estimated with greater statistical power.

However, combining results from epidemiological studies that differ to some degree in their designs and methods of statistical analysis is always somewhat controversial, and here there are several concerns. These probably contribute to the fact that no formal meta-analysis has been carried out to date. First, combining the results of the studies of endogenous oestrogen and breast cancer is not completely straightforward, because of the different ways in which studies have analysed and reported their results. Some report odds ratios between quantiles of oestrogen, some as an odds ratio per s.d. or unit increase in (log transformed) oestrogen, and some only as the mean difference in oestrogen between cases and controls. However, provided assumptions concerning the distribution of oestrogen are satisfied, it is possible (Greenland and Longnecker, 1992; Chene and Thompson, 1996) to estimate odds ratios for a unit increase in a risk factor even when results are presented in one of the other ways described above, and so this type of concern can be dealt with. We describe the methods used to do this in detail under statistical analyses.

Dealing with methodological differences in the designs of the individual studies is more problematic. There is substantial within-subject variation in oestrogen levels across the menstrual cycle, and it is possible that the relationship between oestrogen levels and breast cancer risk differs according to the phase of the cycle. Pooling results from studies conducted using oestrogen levels measured in different phases may obscure this, as may merely adjusting for phase (without taking account of a possible interaction) within the individual studies. Further, the methods used to adjust for phase in the menstrual cycle vary between studies, from simple methods, such as matching on day of cycle or stratifying on phase, to the more sophisticated approach of obtaining residuals from spline regressions of the oestrogen curve throughout the menstrual cycle. In addition, most studies adjust or match for several other breast cancer risk factors, the choice of which varies from study to study. These methodological differences will inevitably introduce a degree of heterogeneity into the results of the studies and, given the available information from each study, this heterogeneity cannot be completely removed through careful statistical analysis. However, despite these differences, we believe that as the studies are all investigating the same basic question, a formal meta-analysis is justified. Such a meta-analysis needs to acknowledge that the differences in study design mean that it is virtually certain that there will be heterogeneity in the magnitude of the observed associations and to quantify this. If the extent of the heterogeneity was large, then this would cast doubt on the validity of a pooled estimate.

We therefore carried out a systematic review of the literature to identify all prospective studies of endogenous premenopausal oestrogens on breast cancer risk. To estimate the effect of a doubling of circulating premenopausal oestrogen levels on the risk of breast cancer, we combined the results from seven prospective studies, which include 693 breast cancer cases. We used a random-effect meta-analysis to take account of the anticipated heterogeneity, resulting from the differences in methodology between studies described above.

## Materials and Methods

### Identification of studies

Studies were identified by performing a literature search on Medline and Embase from 1950 to February 2009, of articles containing the words ‘breast’ in combination with any of the terms ‘cancer’, ‘carcinoma’, ‘neoplasia’ or ‘tumour’ and the terms ‘estrogen’, ‘estriol’, ‘estradiol’, ‘estrone’, ‘hormone’ or ‘steroid’ in the title. Articles were restricted to prospective studies of circulating premenopausal oestrogens on breast cancer risk. The search (carried out by KW) produced 7895 articles, of which 148 titles were identified as potentially eligible. Overall, 13 studies were included based on their abstracts, but 6 were excluded after reading the full-text articles, 2 because the subjects were all postmenopausal, 2 because the cases were all a subset of one of the studies included in the meta-analysis, and 2 studies measured hormones other than oestrogens. The remaining seven studies were all nested matched case–control studies (Wysowski *et al*, 1987; Helzlsouer *et al*, 1994; Rosenberg *et al*, 1994; Thomas *et al*, 1997; Kabuto *et al*, 2000; Kaaks *et al*, 2005; Eliassen *et al*, 2006). In addition, the references of the selected articles and two review articles (Key, 1999; Travis and Key, 2003) were manually searched, but no additional articles were identified. No subjects were included in more than one study; two studies included women from the same cohort (Wysowski *et al*, 1987; Helzlsouer *et al*, 1994), but there was no overlap in participants selected for each study. Matched odds ratios for quantiles or linear increases in log-transformed E2, and all available summary statistics of (log transformed) E2 in cases and controls, were extracted from each study.

### Statistical analyses

The distribution of E2 is positively skewed and a number of the individual studies considered the effect of E2 on breast cancer risk after logarithmic transformation of E2. We took the same approach, and assumed that a log-normal distribution for E2 is reasonable, so as to yield estimates of the odds ratio associated with any multiplicative increase of circulating E2. We present the odds ratio for a doubling of circulating E2. Strictly, odds ratios that are adjusted for (and/or matched on) different sets of covariates are not comparable (Steyerberg and Eijkemans, 2004). However, in most instances such ‘heterogeneity bias’ is small and we judged it preferable to use maximally adjusted estimates in the primary meta-analysis, so as to minimise bias arising through confounding. Where possible, as a check on the robustness of results, adjusted and unadjusted odds ratios were estimated or extracted from the individual studies.

The diversity in the ways in which results were reported meant that a range of techniques were needed in order to estimate the log odds ratio for a doubling in E2 and its standard error in the various studies. Maximally adjusted, matched odds ratios were calculated or extracted from all studies, except for Wysowski *et al* (1987), where such estimates were not reported. The studies by Rosenberg *et al* (1994) and Kaaks *et al* (2005) adjusted for phase in the cycle, using spline regression as well as matching on day of cycle, and we used the matched spline-adjusted estimates from both studies. Rosenberg *et al* (1994) also reported the results of an analysis using serial measurements of E2 on each woman, but we used the results from their analysis of each woman's first measurement of E2 to be consistent with the other studies.

Three studies (Rosenberg *et al*, 1994; Thomas *et al*, 1997; Kabuto *et al*, 2000) reported odds ratios for selected changes in logarithmically transformed E2. Specifically, Thomas *et al* (1997) reported the odds ratio for a unit increase in log_e_(E2), Kabuto *et al* (2000) for a unit increase in log_10_(E2) and Rosenberg *et al* (1994) for a 1 s.d. increase in spline-adjusted log_e_(E2). These estimates (and corresponding standard errors) were used to directly estimate the log odds ratio for a doubling of E2 and its standard error in these studies.

In three further studies (Helzlsouer *et al*, 1994; Kaaks *et al*, 2005; Eliassen *et al*, 2006), results concerning the log odds ratio in each quantile of E2 compared with baseline were used to estimate the log odds ratio for a doubling of E2 and its standard error, using the method described by Greenland and Longnecker (1992). The Greenland and Longnecker (1992) method requires estimates of the mean log_e_(E2) in controls and the overall s.d. of log_e_(E2) in order to estimate the mean of log_e_(E2) in each quantile, as described by Chene and Thompson (1996). The mean and s.d. of log_e_(E2) were estimated as the intercept and slope of normal quantile–quantile plots of the quantiles of log_e_(E2), respectively, separately for cases and controls. The pooled s.d. in cases and controls was used as the overall s.d., after testing for evidence of a difference in s.d. values in cases and controls using an *F* test. The odds ratios reported by Kaaks *et al* (2005) are for quartiles of residuals of E2 from a spline regression model used to model the cyclic change in E2. Quartiles of the residuals in controls added to the mean in controls were assumed to be the quartiles of E2 in controls, adjusted for phase in cycle.

In the study by Wysowski *et al* (1987), adjusted, matched estimates were not presented, and so linear discriminant function analysis (Hand *et al*, 1998) was used to obtain estimates of the log odds ratio, and its standard error (ignoring the matching) from the means of E2 in cases and controls. No measure of spread of E2 was given in this paper. Accordingly, a pooled s.d. from the other six studies was used. Methods used to estimate the s.d. of log_e_(E2) in cases and controls for each study are summarised in Table 2. We pooled the 10 estimates of s.d. of log_e_(E2) across cases and controls and across studies, first pooling the luteal and follicular estimates of s.d. from the study by Eliassen *et al* (2006) separately in cases and controls. The means of E2 in cases and controls, along with this pooled s.d., were used to estimate the mean of log_e_(E2) in cases and controls (Weisstein, 2010), and hence the log odds ratio for a doubling of E2 and its standard error.

The above sections describe the way in which our best estimates of the log odds ratio and its standard error were obtained in each study. In addition, in five of the studies (Helzlsouer *et al*, 1994; Rosenberg *et al*, 1994; Thomas *et al*, 1997; Kaaks *et al*, 2005; Eliassen *et al*, 2006) it was possible to obtain estimates using more than one approach, and we compared these estimates within studies. The sensitivity of the meta-analysis to the choice of estimation method was also examined. This allowed a comparison of matched estimates (using reported matched estimates or by implementing the Greenland and Longnecker (1992) method) and unadjusted estimates ignoring the matching (by implementing Discriminant Function Analysis; Supplementary Table 1).

The individual study estimates were combined using a random-effect meta-analysis, as described by DerSimonian and Laird (1986). A random-effect approach was used, as we anticipated some heterogeneity in study-specific effects due to the methodological differences between studies. The random-effect meta-analysis uses both the variance of the study-specific results and the estimated between-study variance in results to assign weights to the individual studies. This results in more homogeneous weights than a fixed-effect inverse-variance weighted meta-analysis. Between-study heterogeneity was assessed using *I*^2^-statistics and Cochran's *Q*-statistic. Publication bias was explored using funnel plots and Galbraith plots (Galbraith, 1988).

Eliassen *et al* (2006) collected two blood samples from each woman, one from each of the follicular and luteal phases of the menstrual cycle, and reported separate odds ratios for each phase. As oestrogen levels within women are correlated (Missmer *et al*, 2006), the meta-analysis was carried out twice, once including the follicular estimate and once the luteal estimate from this study. Helzlsouer *et al* (1994) stratified their analysis by follicular and luteal phase, with measurements in each phase from different women, and we pooled the two estimates before including them in the meta-analysis.

Two of the studies also reported the association between breast cancer risk and circulating levels of free E2 (Kabuto *et al*, 2000; Eliassen *et al*, 2006), an estimate of the amount of circulating E2 that is unbound to sex hormone binding globulin. The meta-analysis was repeated using the same methods described above to obtain a combined estimate of the odds ratio for breast cancer with a doubling of free E2.

## Results

The seven studies, which provided 693 breast cancer cases and 1609 controls, are summarised in Table 1. Four studies were from US populations (Wysowski *et al*, 1987; Helzlsouer *et al*, 1994; Rosenberg *et al*, 1994; Eliassen *et al*, 2006), two from European populations (Thomas *et al*, 1997; Kaaks *et al*, 2005) and one from a Japanese population (Kabuto *et al*, 2000). The age ranges of the women in the studies were similar, from approximately 25 to 55. Six out of seven studies matched on time in menstrual cycle (Wysowski *et al*, 1987; Helzlsouer *et al*, 1994; Rosenberg *et al*, 1994; Thomas *et al*, 1997; Kaaks *et al*, 2005; Eliassen *et al*, 2006), and all studies matched on some other factors such as demographic or reproductive factors (Table 1), but estimates from the matched analyses were available for only six of the studies. In two of the studies, women were known to be premenopausal at the time of their breast cancer diagnosis (Wysowski *et al*, 1987; Helzlsouer *et al*, 1994). In all but one of the studies (Wysowski *et al*, 1987; Helzlsouer *et al*, 1994; Rosenberg *et al*, 1994; Thomas *et al*, 1997; Kaaks *et al*, 2005; Eliassen *et al*, 2006) it is stated that women using hormonal contraceptives at the time of the blood draw were excluded. Two of the seven studies were limited to invasive breast cancer cases (Rosenberg *et al*, 1994; Kaaks *et al*, 2005).

**Figure 1:**
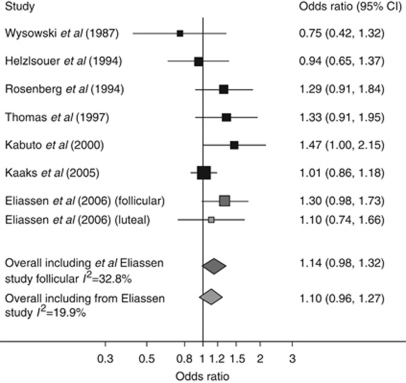
Breast cancer odds ratios for a doubling of endogenous E2 within studies and pooled estimates across studies. Note that the size of each box is proportional to its weight in the meta-analysis that included the follicular estimate from the Eliassen *et al* (2006) study, apart from the light grey box, which has size proportional to its weight in the meta-analysis including the luteal estimate from Eliassen *et al* (2006) study. The relative weights in the meta-analysis including the follicular estimate from Eliassen *et al* (2006) were (in the same order as the graph) 0.06, 0.12, 0.13, 0.11, 0.11, 0.30, 0.17, and for the study including luteal estimate from Eliassen *et al* (2006) were 0.06, 0.12, 0.13, 0.11, 0.11, 0.37, 0.10.

Table 2 reports estimates of the geometric mean and geometric s.d. of E2 in cases and controls, within each study. The pooled geometric s.d. was close to 2 (1.91), indicating that levels 1 s.d. above the (geometric) mean were approximately double the geometric mean, while levels 1 s.d. below the geometric mean were approximately half of it. The estimated odds ratios for a doubling of E2 for each study, with a pooled odds ratio from the random-effect meta-analysis, (a) including the luteal estimate from the study by Eliassen *et al* (2006) and (b) using the follicular estimate from this study, are summarised in Figure 1. There was weak evidence of a positive association between circulating levels of E2 and breast cancer risk, with a doubling of E2 conferring a 10% (95% CI: −4, 27; *P*=0.08) to 14% (95% CI: −2, 32; *P*=0.17) increase in the odds of developing breast cancer, depending on which estimate is included from the Eliassen *et al* (2006) study. Assuming that E2 is approximately log-normally distributed with geometric s.d. 1.91, our more conservative estimate equates to distributions in cases and controls 0.091 (95% CI: −0.039, 0.222) s.d. values apart and hence to an odds ratio of 1.26 (95% CI: 0.90, 1.76) comparing women in the top and bottom quartiles of E2. Table 3 reports estimated odds ratios for a selection of percentage increases in E2.

The combined estimated odds ratio for a doubling of free E2 (from the two studies that provided estimates) was 1.27 (95% CI: 1.00, 1.61) or 1.41 (95% CI: 1.13, 1.74) when including the luteal and follicular estimates from Eliassen *et al* (2006) study, respectively. For comparison, the equivalent estimates for total E2 for the same two studies were 1.29 (95% CI: 0.97, 1.70) or 1.36 (95% CI: 1.08, 1.70), respectively.

There was little evidence of between-study heterogeneity, with the proportion of variability between studies falling between 20% and 33%, depending on which estimate was included from the study by Eliassen *et al* (2006). The funnel plots and Galbraith plots showed no evidence of publication bias. The results of the meta-analysis were not substantially affected by the methods used to estimate the dose–response effect estimates: the pooled random-effect estimate from unadjusted estimates ignoring the matching, for a doubling of E2, where available (using Discriminant Function Analysis in six of the studies and the reported unadjusted matched estimate from the study by Kabuto *et al* (2000)), was 1.08 (95% CI: 0.98, 1.20) and 1.12 (95% CI: 1.00, 1.26), when including the luteal and follicular measurements from Eliassen *et al* (2006) study, respectively. Within studies, the unadjusted odds ratio ignoring the matching was very close to the adjusted matched odds ratio for four out of five of the studies where it was possible to estimate odds ratios using two methods (Supplementary Table 1).

## Discussion

Our analysis provides some evidence of a positive association between circulating E2 levels and breast cancer risk in premenopausal women. We estimate that a doubling of circulating E2 increases a woman's risk of breast cancer by 10% (95% CI: −4, 27).

The breast cancer odds ratio for a doubling of postmenopausal E2 has been estimated to be 1.29 (95% CI: 1.15, 1.44; The Endogenous Hormones and Breast cancer Collaborative Group, 2002), which is larger than our estimate for premenopausal E2. The confidence intervals overlap, however, and it is therefore still unclear whether there is a different effect of circulating E2 in women before and after the menopause. Within-subject variability in E2, which is likely to be substantially larger in premenopausal than postmenopausal women, will dilute the effect estimate, and this could contribute to the difference in the estimates. The majority of breast cancer cases in the meta-analysis are likely to be premenopausal, and there may be differences in the aetiology of premenopausal and postmenopausal breast cancer, which result in oestrogen levels influencing breast cancer risk to a lesser extent before the menopause. For example, premenopausal breast cancers are more likely to be oestrogen receptor negative and therefore not sensitive to oestrogen (Vasiljevic *et al*, 1998). Perhaps circulating premenopausal oestrogen has a different effect on breast cancer risk than circulating postmenopausal oestrogen; premenopausal E2 is produced predominantly in the ovaries, while in postmenopausal women circulating levels are much lower, and production is predominantly by conversion of androgen precursors in adipose tissue into oestrone, which is then converted into E2 (Travis and Key, 2003). We estimated the geometric s.d. of E2 to be 1.91 in premenopausal women, while the geometric s.d. in postmenopausal women, estimated from the lower and upper quartiles of E2 in the published pooled analysis (The Endogenous Hormones and Breast cancer Collaborative Group, 2002) using a Q–Q plot, is 1.67. This is consistent with within-subject variability being somewhat greater in pre- than postmenopausal women, but nonetheless the total variability on the multiplicative scale appears to be sufficiently similar for informal comparisons of the effect of a doubling of levels to be made.

The endogenous androgens testosterone, androstenedione, DHEA and DHEAS, which do not have the cyclical variation of oestrogen, have been found to have similar effects on breast cancer risk in premenopausal and postmenopausal women. Odds ratios for breast cancer for premenopausal women in the top *vs* bottom quartiles of several androgens in the study by Kaaks *et al* (2005) range from 1.5 to 1.7 , while for postmenopausal women in the top *vs* bottom quintiles of the same androgens, the estimates range from 1.7 to 2.2 (The Endogenous Hormones and Breast cancer Collaborative Group, 2002). This supports the argument that one contributory factor to the difference in estimates for premenopausal and postmenopausal E2 could be increased within-subject variability before the menopause.

Rosenberg *et al* (1994), however, report an estimate using the mean of several E2 measurements from the same women, thus reducing measurement error in the exposure. This result, which was not included in our meta-analysis, shows a similar association between oestrogen and breast cancer risk to their estimate using a single E2 measurement, whereas a stronger association would be expected if measurement error really was biasing the result towards the null.

All studies used E2 as the exposure, and in addition oestriol was measured in one study (Wysowski *et al*, 1987) and four studies measured oestrone (Wysowski *et al*, 1987; Helzlsouer *et al*, 1994; Kaaks *et al*, 2005; Eliassen *et al*, 2006). E2 was chosen as the exposure for the meta-analysis because it is the predominant oestrogen in premenopausal women (Herjan, 2004), and was measured in all seven studies. The estimates of the effects of circulating oestrone on breast cancer risk were similar to those of circulating E2 in the four studies, in which both oestrogens were measured, albeit with somewhat larger effect estimates for luteal oestrone than luteal E2 (but similar estimates for follicular oestrone and E2) in the study by Eliassen *et al* (2006).

All studies included in the meta-analysis matched cases and controls. All estimates in the primary meta-analysis are from fully matched/adjusted analyses, apart from those from the study by Wysowski *et al* (1987). However, different studies match and adjust for different factors, including measures of adiposity, reproductive factors and family history of breast cancer. One study reported adjusted and unadjusted (matched) estimates (Eliassen *et al*, 2006) and found that estimates adjusting for BMI at the age of 18 years, age at menarche and first birth, parity, history of benign breast disease and family history of breast cancer, were only slightly larger than unadjusted (matched) estimates. We also found that within studies our estimate of the odds ratio ignoring the matching and adjustment was very close to the adjusted matched odds ratio for all studies where we were able to estimate both, except for the study by Rosenberg *et al* (1994), in which matching and adjustment changed the estimate from close to one to a modest, but nonsignificant association. Carrying out a meta-analysis where we used estimates ignoring matching and adjustment (where possible) gave very similar results to those where we did allow for matching and adjustment. These findings, together with the reasonably low between-study heterogeneity, imply that our combined estimates are reasonably robust to differences in matching and adjustment between studies.

We assumed in our analysis that a log-normal distribution for E2 is reasonable. In three of the studies (Rosenberg *et al*, 1994; Thomas *et al*, 1997; Kabuto *et al*, 2000) it is explicitly stated that E2 was log-normally distributed. In addition, the 12.5th and 87.5th percentiles of E2, reported by Eliassen *et al* (2006) study, are equidistant from their reported median, after log transformation, which means that log transformation removes the positive skew observed in their data. Similarly, the 25th and 75th percentiles of the residuals of E2 in controls from the spline regression carried out by Kaaks *et al* (2005) are equidistant from their median after log transformation. It seems therefore that a log-normal assumption is reasonable.

It is possible that the effect of a doubling of E2 on breast cancer risk differs according to the phase of the menstrual cycle. This, coupled with the fact that different methods were used to match or adjust for time of blood sample collection in the menstrual cycle in the various studies, may limit the utility of a single pooled estimate. However, a number of factors suggest that our pooled estimate may well be generalisable to the different phases of the cycle. First, we did not find any statistically significant evidence of heterogeneity in the magnitude of associations across studies. Second, although one study (Eliassen *et al*, 2006) did report weak evidence of an association in the follicular phase, but not in the luteal phase, confidence intervals on the magnitude of the associations in the two phases were wide and they did not report the result of a statistical interaction test for a difference between these (Matthews and Altman, 1996). Third, in a previous study (Walker *et al*, 2009) we took repeated measurements of urinary oestrone glucuronide, a principal metabolite of serum oestrogens that is highly correlated with serum E2 (Tanabe *et al*, 2001), in premenopausal women, and estimated the association at different times in the menstrual cycle with mammographic density, a very strong marker of breast cancer risk. We found that the mean level of oestrone glucuronide throughout the cycle is the most biologically relevant measure associated with mammographic density, rather than that in any particular phase in the cycle. This provides us with some evidence that our pooled results are not materially affected by the different ways in which time in the menstrual cycle was accounted for.

In postmenopausal women, circulating levels of free E2 were found to have a larger effect on breast cancer risk than total E2 (The Endogenous Hormones and Breast cancer Collaborative Group, 2002). We estimated a similar increased risk in breast cancer for a doubling of free E2 and a doubling of total E2; however, there was a large amount of uncertainty in both estimates as only two studies provided estimates of the association with free E2. We have no evidence to suggest that circulating levels of free E2 are more strongly associated with breast cancer risk than total E2 in premenopausal women.

Helzlsouer *et al* (1994) and Eliassen *et al* (2006) both report results stratifying by phase in the menstrual cycle, and both found a stronger association with breast cancer in the follicular than the luteal phase. The study by Thomas *et al* (1997) showed that the difference in E2 between cases and controls was greatest in the mid-cycle, at the transition between the follicular and luteal phases, but this was based on only seven cases in the mid-cycle. It remains to be seen whether E2 levels at a particular point in the cycle are more important in the aetiology of breast cancer than the average level over time.

It is worth considering whether a woman's circulating E2 level could be added to models for projecting the risk of breast cancer, such as the Gail model or the ‘Gail model 2’ (Costantino *et al*, 1999). Gail (2008) demonstrated that adding seven single-nucleotide polymorphisms to the ‘Gail model 2’, each of which confers an OR of between 1.07 and 1.26 and has a carrier frequency of between 0.13 and 0.50, added very little discriminatory accuracy to the prediction model. If premenopausal women in the top quartile of circulating E2 carry an increased risk somewhere in the region of 25% compared with those in the bottom quartile, adding E2 level to the model would do little to improve its prognostic accuracy.

This meta-analysis has demonstrated weak evidence of a positive association between premenopausal endogenous E2 levels and breast cancer risk. More studies are needed to accurately quantify this association, in order to provide meaningful estimates and to allow a comparison with the association in postmenopausal women. Repeated measurements in each woman may be helpful to reduce measurement error in E2. Further estimates in the follicular and luteal phases of the cycle separately would aid the discussion of whether E2 in a particular phase in the cycle is important, or whether mean levels across the cycle are implicated.

## Figures and Tables

**Table 1:** Summary of the characteristics and main findings of the studies in the meta-analysis

**Study**	**Cases/controls**	**Age range (years)**	**Cohort**	**Effect estimate from main analysis**	**Main result (95% CI)** a	**Matched or adjusted for time in cycle?**	**Additionally adjusted for/matched on**
Wysowski *et al* (1987) b	17/67	25–50	Washington County	Mean difference in matched cases *vs* controls	Mean difference not reported (*P* for difference > 0.05)	Matched to exact days since last cycle	Age, race
							
Helzlsouer *et al* (1994)	22/44	25–54	Washington County	OR for above *vs* below median E2	0.7 (0.2, 3.1)	Matched to within 1 day since last cycle + luteal and follicular samples analysed separately	Age, race, time of day of blood draw, interval between last meal and blood draw
							
Rosenberg *et al* (1994)	79/306	34–55	NYU Women's Health Study	OR for 1 s.d. increase in residual log_e_E2 from spline regression	1.19 (0.91, 1.55)	Matched to exact day since last cycle + spline adjusted	Age, date of blood draw, no. of blood samples
							
Thomas *et al* (1997)	61/179	33–49^c^	Guernsey	OR for 1 unit increase in log_e_ E2	1.51 (0.87, 2.63)	Matched to within 2 days to next cycle	Age, parity, first-degree family history breast cancer, BMI, past OC use, other hormone concentrations, date of blood draw
							
Kabuto *et al* (2000)	46/94	NA	Atom bomb survivors Hiroshima and Nagasaki	OR for 1 unit increase in log_10_E2	3.6 (1.1, 13.4)	Not matched or adjusted for	Age, city, date of blood draw, radiation dose
							
Kaaks *et al* (2005)	283/551	37–53^c^	EPIC	OR for quartiles of residual E2 from spline regression	1.0 (0.66, 1.52)	Matched on categories of days to next/last cycle + spline adjusted	Age, study centre, BMI, age first pregnancy, age menarche, no. children, time of day of blood draw
							
Eliassen *et al* (2006)	185/368	37–50^c^	Nurses' Health Study II	OR for quartiles of E2	Follicular phase: 2.1 (1.1, 4.1), luteal phase: 1.0 (0.5, 1.9)	Adjusted for days from last cycle (follicular samples) or days to next cycle) luteal samples) + luteal and follicular samples analysed separately	Age, BMI at the age of 18 years, age at menarche, age first birth, parity, history benign breast disease, family history breast cancer, ethnicity, time of day of blood draw

Abbreviations: BMI=body mass index; CI=confidence interval; NA=not applicable; OC=oral contraceptive; OR=odds ratio.

a Where OR for quantiles of E2 were reported, the result is for the top *vs* bottom quantile.

b Although their analysis was matched, only unmatched estimates were presented in the paper.

c Indicates 5th to 95th percentile.

**Table 2:** Estimated geometric mean and geometric s.d. E2 in cases and controls for each study, and the estimation methods used

	**Estimated geometric mean (s.d.) E2**		
**Study**	**Cases**	**Controls**	**Method used to estimate s.d. log_e_(E2) in cases and controls**
Wysowski *et al* (1987)	84.8 (1.91)	101.5 (1.91)	Pooled s.d. across cases and controls from all other studies
Helzlsouer *et al* (1994)	44.7 (3.00)	47.9 (NA)	Two quantiles in cases (median in cases plus a further quantile calculated from no. of cases above and below control median) on a Q–Q plot used to estimate s.d. E2 in cases. s.d. log_e_(E2) calculated from mean and s.d. E2, assuming a normal distribution for log_e_(E2)
Rosenberg *et al* (1994)	137.0 (2.19)	138.4 (2.01)	s.d. log_e_(E2) in cases and controls reported
Thomas *et al* (1997) a	86.0 (1.72)	77.0 (1.72)	Geometric mean E2 in cases and controls, with 95% CIs, reported
Kabuto *et al* (2000)	NA	87.4 (2.03)	s.d. log_10_(E2) in controls reported
Kaaks *et al* (2005) a	86.3 (1.97)	80.5 (2.03)	s.d. log_e_(E2) calculated from mean and s.d. E2, assuming a normal distribution for log_e_(E2), separately for cases and controls
Eliassen *et al* (2006) (follicular)	49.4 (1.73)	43.8 (1.82)	Quartiles plus median, 12.5th and 87.5th percentiles E2 on a Q–Q plot used to estimate mean and s.d. E2. s.d. log_e_(E2) calculated from mean and s.d. E2, assuming a normal distribution for log_e_(E2), separately for cases and controls
Eliassen *et al* (2006) (luteal)	120.3 (1.43)	117.9 (1.55)	As above, separately for luteal E2

Abbreviations: CIs=confidence intervals; NA=not applicable; Q–Q=quantile–quantile plot.

a Thomas *et al* and Kaaks *et al* report the mean E2 in pmol l^−1^. These were converted to pg ml^−1^ by multiplying by 0.272.

**Table 3:** Estimated ORs for a range of percentage increases of circulating E2

	**Including luteal measurements from Eliassen *et al* study**		**Including follicular measurements from Eliassen *et al* study**	
**Increase in E2**	**OR**	**(95% CI)**	**OR**	**(95% CI)**
10%	1.01	(0.99, 1.03)	1.02	(1.00, 1.04)
20%	1.03	(0.99, 1.06)	1.04	(1.00, 1.08)
50%	1.06	(0.98, 1.15)	1.08	(0.99, 1.18)
**100% (doubling)**	1.10	(0.96, 1.27)	1.14	(0.98, 1.32)
400%^a^	1.25	(0.91, 1.73)	1.36	(0.96, 1.91)

Abbreviations: CI=confidence interval; OR=odds ratio.

a Corresponds approximately to a woman in 90th percentile E2 compared with a woman in 10th percentile.
