# Supplementary material for: Premenopausal endogenous oestrogen levels and breast cancer risk: a meta-analysis
Source: Br J Cancer. 2011 Sep 13;105(9):1451–7. doi: 10.1038/bjc.2011.358 (PMC3241538; doi:10.1038/bjc.2011.358)
Supplement: Supplementary Information [file bjc2011358x1.doc]

**Supplementary table 1:** Comparison of estimates using different estimation methods. The “matched adjusted” analysis is the main meta-analysis.

|  | **"Matched adjusted" analysis** | | **"Unmatched unadjusted" analysis** | |
| --- | --- | --- | --- | --- |
| **Study** | **Estimation method** | **Odds ratio (95% CI)** | **Estimation method** | **Odds ratio (95% CI)** |
| Wysowski et al 1987 | - | - | DFA | 0.76 (0.43,1.32) |
| Helzlsouer et al 1994 | G-L | 0.94 (0.65,1.37) | DFA | 0.96 (0.70,1.33) |
| Rosenberg et al 1994 | Reported matched adjusted OR | 1.29 (0.91,1.84) | DFA | 0.99 (0.78,1.25) |
| Thomas et al 1997 | Reported matched adjusted OR | 1.33 (0.91,1.95) | DFA | 1.30 (0.90,1.89) |
| Kabuto et al 2000 | Reported matched adjusted OR | 1.47 (1.00,2.15) | Reported matched adjusted OR | - |
| Kaaks et al 2005 | G-L | 1.01 (0.86,1.18) | DFA | 1.10 (0.95,1.26) |
| Eliassen et al 2006 (follicular) | G-L | 1.30 (0.98,1.73) | DFA | 1.29 (1.05,1.59) |
| Eliassen et al 2006 (luteal) | G-L | 1.10 (0.74,1.66) | DFA | 1.08 (0.80,1.46) |
